# RNA methylation pattern and immune microenvironment characteristics mediated by m6A regulator in ischemic stroke

**DOI:** 10.3389/fgene.2023.1148510

**Published:** 2023-04-17

**Authors:** Kejuan Jia, Wenbo Xia, Qian Su, Shiqi Yang, Yanli Zhang, Xunran Ni, Zhiqiang Su, Delong Meng

**Affiliations:** ^1^ The First Affiliated Hospital of Harbin Medical University, Harbin, Heilongjiang, China; ^2^ The Key Laboratory of Myocardial Ischemia, Harbin Medical University, Ministry of Education, Harbin, Heilongjiang, China; ^3^ Heilongjiang Provincial Hospital, Harbin, Heilongjiang, China

**Keywords:** ischemic stroke, m6A, m6A modification pattern, immune microenvironment, machine learning

## Abstract

**Background:** Ischemic stroke (IS) is a highly heterogeneous disease. Recent studies have shown that epigenetic variables affect the immune response. However, only a few studies have examined the relationship between IS and m6A immunoregulation. Therefore, we aim to explore the methylation of RNA mediated by m6A regulatory factor and the immune microenvironment characteristics of IS.

**Methods:** Differentially expressed m6A regulators were detected in IS microarray datasets GSE22255 and GSE58294. We used a series of machine learning algorithms to identify key IS-related m6A regulators and validated them on blood samples of IS patients, oxygen-glucose deprivation/reoxygenation (OGD/R) microglia and GSE198710 independent data sets. Different m6A modification modes were determined and the patients were classified. In addition, we systematically associate these modification patterns with the characteristics of immune microenvironment, including infiltrating immune cells, immune function genes and immune response genes. Then we developed a model of m6A score to quantify the m6A modification in IS samples.

**Results:** Through the analysis of the differences between the control group and IS patients, METTL16, LRPPRC, and RBM15 showed strong diagnostic significance in three independent data sets. In addition, qRT-PCR and Western blotting also confirmed that the expression of METTL16 and LRPPRC was downregulated and the expression of RBM15 was upregulated after ischemia. Two m6A modification modes and two m6A gene modification modes were also identified. m6A gene cluster A (high m6A value group) was positively correlated with acquired immunity, while m6A gene cluster B (low m6A value group) was positively correlated with innate immunity. Similarly, five immune-related hub genes were significantly associated with m6Acore (CD28, IFNG, LTF, LCN2, and MMP9).

**Conclusion:** The modification of m6A is closely related to the immune microenvironment. The evaluation of individual m6A modification pattern may be helpful for future immunomodulatory therapy of anti-ischemic response.

## 1 Introduction

Ischemic stroke (IS) accounts for 80% of all stroke cases and has a very high rate of disability (Iadecola et al., 2020). Post-stroke inflammation may exacerbate brain injury for a longer period, and Fang et al. found that targeting post-stroke inflammation may provide a later and broader window of potential treatment than thrombolysis or mechanical thrombectomy (Zhang et al., 2021). Considering the complexity, dynamics, and prolongation of inflammation after IS, neuroimmune-targeted therapies have not been clinically applied until recently. A recent study found that modulating post-transcriptional RNA levels using RNA-binding proteins, and epigenetic post-transcriptional modifications may be an effective treatment for post-ischemic pathophysiology (Yao et al., 2020). In light of these observations, abnormal m6A modifications could play a key role in ischemic cascades, in which neurogenesis, glutamate-mediated excitability is involved, and in secondary brain injury after IS (Yao et al., 2020). Therefore, epigenetic regulation in IS has received considerable attention.

N6-methyladenosine (m6A) is the most abundant mRNA chemical modification, especially in the brain (Yen and Chen, 2021). Proteins involved in m6A regulation include “writers” (m6A methyltransferases), “erasers” (m6A demethylases), and “readers” (m6A binding proteins); collectively, they support a dynamic balance between mRNA methylation and demethylation (Yang et al., 2018). Methyltransferase complexes, including METTL3, METTL14, and WTAP, can induce RNA methylation. Demethylases such as obesity-associated protein (FTO) and ALKB homolog 5 (ALKBH5) are primarily responsible for reversing RNA methylation. m6A binding proteins, such as YTHDC1/2 and IGF2BP1/2/3, recognize and bind substrate RNA to perform their functions (Patil et al., 2016; Chen et al., 2019; He and He, 2021; Liu et al., 2023). Nevertheless, when these regulatory factors are abnormal, they affect multiple aspects of mRNA structure, alternative splicing, translation, nucleation, stability, and immunogenicity, leading to abnormal immune regulation (Wang et al., 2020; Song et al., 2021), dysregulation of cell proliferation (Li et al., 2017), metabolic disorders (Yang et al., 2018), developmental defects (Xu et al., 2020), and tumor progression (Wang et al., 2020). Previous studies have shown that METTL3 promotes the maturation of miR-335 and reduces apoptosis in damaged neurons (Si et al., 2020). ALKBH5 knockout exacerbated neuronal damage and death, whereas FTO overexpression was neuroprotective (XK Mo Y. et al., 2020). Non-etheless, there has been no systematic analysis of m6A RNA modifications involved in the various mechanisms of IS.

In previous studies, it has been demonstrated that methylation modifications can affect immune cell maturation and response. FTO expression was decreased in both M1 and M2 macrophages (Gu et al., 2020). The expression of METTL3 promotes polarization of M1 macrophages, but inhibits polarization of M2 macrophages (Liu et al., 2019). Li et al. (2022a) found that deleting METTL3 in naive T cells reduced the proportion of Th1 and Th17 cells, increased the proportion of Th2 cells, but did not affect Treg cells. Tong et al. (2018) reported that conditional METTL3 knockout in mice resulted in the loss of Treg suppression, leading to the development of severe systemic autoimmune disease. Additionally, METTL3 promotes the transcription of CD40, CD80, and IL-12 in dendritic cells, further inducing the proliferation and differentiation of naive T cells in the adaptive immune system (Wang et al., 2019a). Therefore, when methylation affects different RNAs or cells, it directly or indirectly affects immunity. There is evidence that RNA methylation plays a dual role in tumor immunity (Song et al., 2021); however, it remains unclear how m6A RNA regulators affect immune responses in IS.

There is increasing evidence regarding the role of m6A modifications in immune responses and the relationship between m6A modification interactions between host cells and IS (Si et al., 2020; XK Mo Y et al., 2020; Passaro et al., 2021; Yi et al., 2021). However, current research has mostly focused on a single m6A-related gene and cell type, which may not adequately reflect the reality of m6A methylation modification in immune cells of patients with IS. Consequently, a deeper understanding of m6A modification profiles in immune cells, as well as the interaction between immune cell regulation and m6A modification profiles, is therefore urgently needed.

In this study, we systematically characterized the immune status of controls versus IS samples, as well as the relationship between m6A modification and immune phenotypes.

## 2 Methods

### 2.1 IS dataset sources and preprocessing

From the GEO database, we downloaded two array expression profiling datasets, GSE22255 (Krug T et al., 2011) and GSE58294 (Stamova BB et al., 2014). The platform was GPL570. We downloaded the original “CEL” file and cleaned the data using the “oligo” package and the “removeBatchEffect” algorithm (Ritchie et al., 2015). GSE198710 is an external dataset on the GPL21827 platform. Only mRNA expression profiles were analyed based on the annotation file. Genes with multiple probes were randomly deduplicated to generate individual values.

### 2.2 Acquisition of m6A regulators and related pathways

Based on previous research (Du et al., 2021; Qiu et al., 2021), 26 m6A regulators were identified, and their expression was analyzed using an integrated dataset. Using the “limma” R package, we determined DEGs associated with m6A. Further, *p*-values <0.05 and |logFC| >0.585 were considered statistically significant. Constructing protein-protein interaction networks and m6A regulator-related pathways with Cytoscape.

### 2.3 Acquisition of m6A RNA methylation targets and immune-related genes

The target genes were obtained from the m6A2Target database (http://m6a2target.canceromics.org), which contains experimentally validated and potential m6A modifying enzyme targets. Based on the ImmPort database (https://www.immport.org), immune-related mRNAs (IRs) comprising 1,380 genes were studied. For all data, we selected the mRNA expression profiles of the DEGs obtained for further analysis.

### 2.4 Gene set variation analysis (GSVA) and functional annotation

Considering the molecular biological differences between the groups, we used gene ontology (GO) functional analysis, and the gene set “c2. cp.kegg.v7.5.1. symbols.gmt” obtained from the MSigDB database (http://www.broadinstitute.org/gsea/msigdb/index.jsp) was used for the Kyoto Encyclopedia of Genes and Genomes (KEGG) pathway enrichment analysis (https://www.kegg.jp).

### 2.5 Analysis of characterization of the immune microenvironment

The single-sample gene enrichment analysis (ssGSEA) in the R package “GSVA” was utilized to assess and calculate immune cells, immune function, and immune response in each sample (Bhattacharya et al., 2014). Genome sequences of infiltrating immune cells, immune function genes and immune response genes were obtained from the ImmPort database.

### 2.6 Screening and verification of diagnostic markers

To identify diagnostic markers of IS, we used least absolute shrinkage and selection operator (LASSO) logistic regression and random forest (RF) algorithm to select features. Lambda parameter tuning was done using cross-validation. The cut-off value was determined using ranking statistics. The RF algorithm was performed using the “randomForest” package (https://www.stat.berkeley.edu/∼breiman/RandomForests/). The final step was to combine the results of the two algorithms.

### 2.7 Construction of a nomogram model

We constructed a nomogram model in R using the “rms” package to determine the prevalence of patients with IS. We used calibration curves to demonstrate agreement between the model and cohort data and decision curve analysis (DCA) and clinical impact curves to determine whether the decisions based on the model were beneficial to patients (Iasonos et al., 2008).

### 2.8 Unsupervised clustering for m6A regulators

The “ConsensusClusterPlus” package was used to identify the different m6A modification patterns (Wilkerson and Hayes, 2010). Based on the clustering effect, corresponding heat maps were generated. To observe the distribution of gene expression and verify the signature scores, each cluster underwent principal component analysis (PCA).

### 2.9 Establishment of m6A score

To quantify m6A modification levels, we constructed a new scoring system using m6A-related DEGs, resulting in what we termed as ‘the m6A score’. First, we performed PCA to extract the PC1 and PC2, following which the m6A score was calculated using the following formula: m6A score = ∑(PC1i + PC2i), where i represents each m6A-related gene.

### 2.10 Patient selection and blood samples

From November 2021 to April 2022, we collected blood samples from 40 patients with IS and 40 healthy individuals attending our hospital. Inclusion/exclusion criteria were as follows: 1) Onset of disease within 48 h; 2) Be aged 50–70 years and may have combined hypertensive disease and/or diabetes mellitus; 3) The diagnostic criteria for acute ischemic stroke were met, with clear symptoms of focal neurological deficit and supported by imaging such as brain MRI or CT; 4) signed informed consent. Combination of significant systemic diseases was excluded. Fresh specimens were rapidly frozen and stored in liquid nitrogen.

### 2.11 Cell culture and oxygen glucose deprivation/reperfusion (OGD/R)

HMC3 cells were grown in supplemented medium containing 10% fetal bovine serum, Minimum Essential Medium and 1% penicillin/streptomycin under typical culture conditions (37°C, 5% CO_2_). The standard medium for cells in the plates was discarded and washed twice with PBS, and then transferred to a chamber for 3 h after adding serum-free MEM (5% CO_2_, 95% N_2_). Cells were subsequently transferred to complete medium in a normoxic environment and continued to incubate for a certain period of time to simulate *in vitro* brain ischemia-reperfusion injury.

### 2.12 RNA isolation and qRT-PCR

According to the conventional RNA extraction protocol used in our laboratory, Total RNA was extracted from blood using Trizol LS reagent (10296010, Invitrogen). cDNA was prepared using the Revertra ACE QPCR RT kit (FSQ-101, Toyobo) and qRT-PCR was performed using SYBR Green Master (Roche). All qRT-PCR experiments and data analysis steps were required to comply with to MIQE guidelines. Primer sequences are listed in Table 1.

**TABLE 1:** Primer sequences.

Gene	Forward 5′to 3′	Reverse 3′to 5′
METTL16	TGG​AGC​AAC​CTT​GAA​TGG​CTG​G	CCA​TCA​GGA​GTG​TCT​TCT​GTG​G
LRPPRC	ATC​CGA​CAT​GGT​TAC​TGG​TGG​C	GTG​TCA​AGG​ACA​GCA​GAT​GAA​TC
RBM15	CTT​CCC​ACC​TTG​TGA​GTT​CTC​C	CTT​CTT​GTT​CTC​ATA​CCT​AAC​TCC

### 2.13 Immunoblotting

HMC3 cells were lysed using RIPA Lysis buffer (abs9229, absin). The same amount of proteins were separated by 10% sodium dodecyl sulfate-polyacrylamide gel electrophoresis (SDS-PAGE) and then transferred to PVDF membrane (88518, Thermo Fisher Scientifi). The PVDF membrane was blocked with 5% skim milk powder and then incubated overnight at 4°C with primary antibodies against METTL16 (1:500, 19924-1-AP, Proteintech), LRPPRC (1:20,000, 67679-1-Ig, Proteintech), and RBM15 (1:8,000, 10587-1-AP, Proteintech) and GAPDH (1:10,000, 10494-1-AP, Proteintech). Subsequently, PVDF membranes were incubated with anti-rabbit lgG HRP-linked antibody (1:15,000, 926–32211, Li-Cor) or anti-mouse lgG HRP-linked antibody (1:15,000, 926–68070, Li-Cor) for 1 h at room temperature. Images are acquired using an enhanced chemiluminescent solution under a BIO-RAD workstation.

### 2.14 Statistical analysis

We used the Wilcoxon test to compare differences between groups. Spearman’s test was used for correlation analysis and calculation of correlation coefficients. Statistical significance was determined by *p*-values <0.05. All data were normalized and analyzed using R 4.1.3 and R Bioconductor packages.

## 3 Results

### 3.1 Distribution of m6A regulators between controls and IS samples

**FIGURE 1:**
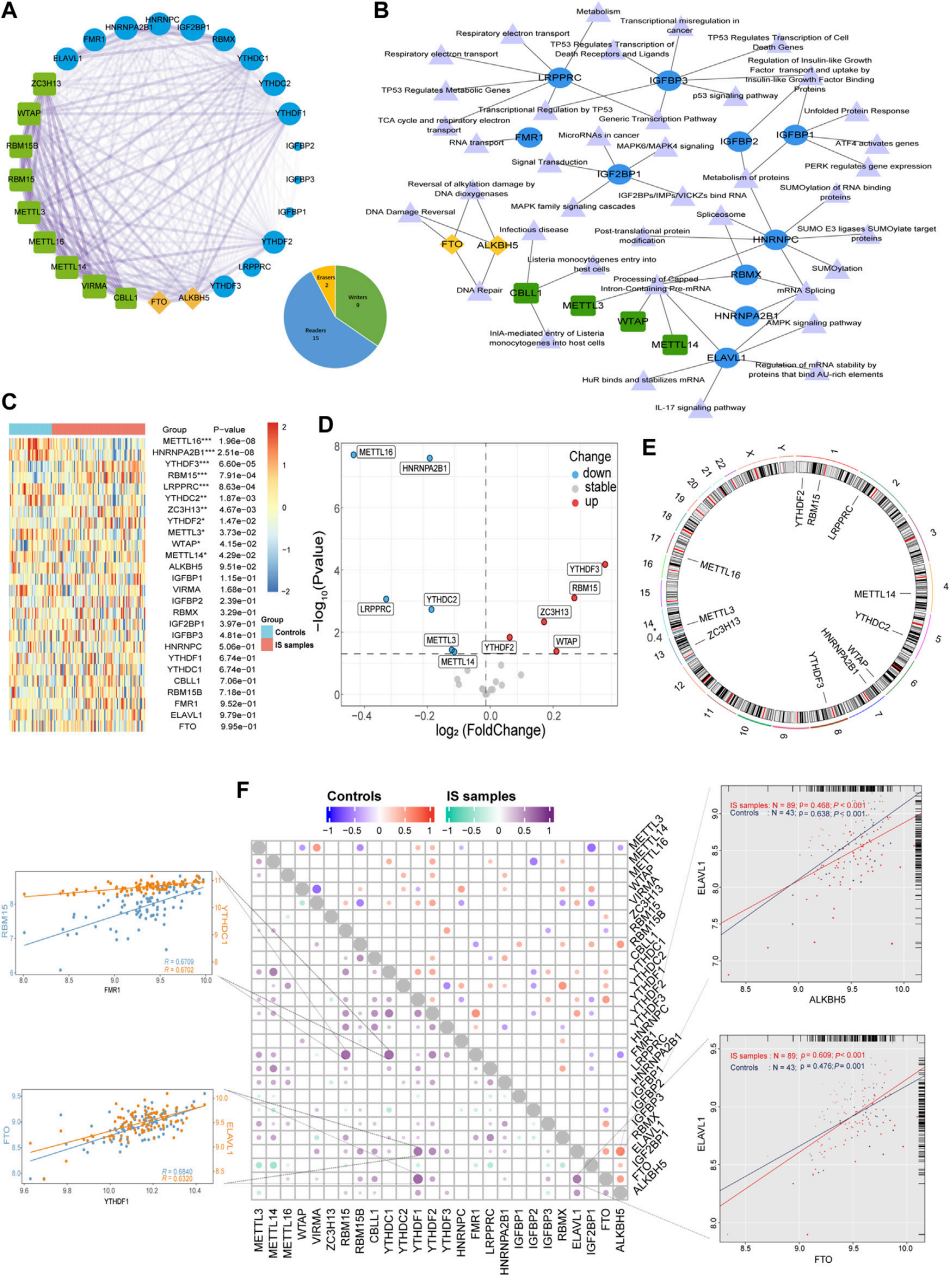
The synergistic effects not only existed in the same type of m6A regulators but also between different types of m6A regulators. **(A)** The composition summary of m6A regulators and the PPI interactions among 26 m6A regulators. **(B)** m6A regulator-pathway interaction network. **(C)** Expression heatmap of the 26 m6A regulators. **(D)** Volcano plot of the expression differences of 26 m6A regulators. **(E)** Chromosomal positions of the differential m6A regulators. **(F)** Dot-plot demonstrating the correlations between 26 m6A regulators in controls and IS samples. The four respective scatterplots show the highest correlation between m6A regulators, the most significant positive correlation of FMR1 with RBM15 and YTHDC1, and the most significant positive correlation of YTHDF1 with FTO and ELAVL1, the most significant positive correlation of FMR1 with RBM15 and YTHDC1, ELAVL1, and ALKBH5 with the most significant positive correlation in controls, ELAVL1 and FTO with the most significant positive correlation in IS samples. *p < 0.05, **p < 0.01, and ***p < 0.001, “ns” indicates no significance.

In this study, 26 recognized m6A regulators were identified, including 9 writers, 15 readers, and 2 erasers. PPI network analysis revealed that the 26 regulators were closely related, and especially that writers often perform complex functions, closely related to both readers and erasers (Figure 1A). The regulator-pathway interaction network revealed that m6A regulators were associated with DNA damage and repair, mRNA stability, protein modification, and cancer signaling (Figure 1B). Before analyzing the data, we merged the different datasets to remove batch effects and then performed a PCA analysis showing that the principal components were separated between the control and IS samples (Supplementary Figure S1). Analysis of the expression differences between IS samples and controls revealed that 11 m6A regulators were significantly associated with IS (Figures 1C, D). Compared with the controls, there were five upregulated genes and six downregulated genes in the IS samples. Two readers (YTHDF2 and YTHDF3) and three writers (RBM15, ZC3H13, and WTAP) were upregulated. Further, three writers (METTL16, METTL3, and METTL14) and three readers (HNRNPA2B1, LRPPRC, and YTHDC2) were found to be downregulated. YTHDF3 and METTL16 were the most significantly upregulated and downregulated genes, respectively. There were significant changes in well-studied writers (METTL3 and METTL14) and readers (YTHDF2 and YTHDF3). RCircos revealed the chromosomal locations of 11 differential m6A regulators (Figure 1E). Moreover, we described the correlation between the three types of m6A regulators and found surprisingly strong synergistic effects between not only the same type of m6A regulators but also between different types of m6A regulators (Figure 1F). For example, in IS samples, YTHDF1 was significantly positively correlated with both ELAVL1 and FTO, whereas FMR1 was significantly positively correlated with both YTHDC1 and RBM15. Moreover, ELAVL1 was strongly correlated with ALKBH5 in controls (*r* = 0.638 in controls and *r* = 0.468 in IS samples). On the other hand, ELAVL1 was strongly correlated with FTO in IS (*r* = 0.476 for controls and *r* = 0.609 for IS samples). The abovementioned results demonstrate that m6A regulator expression was significantly correlated with writers, erasers, and readers in the same functional class, with mostly positive correlations, providing a theoretical foundation for future experiments.

### 3.2 Identification of key m6A regulators in IS samples

**FIGURE 2:**
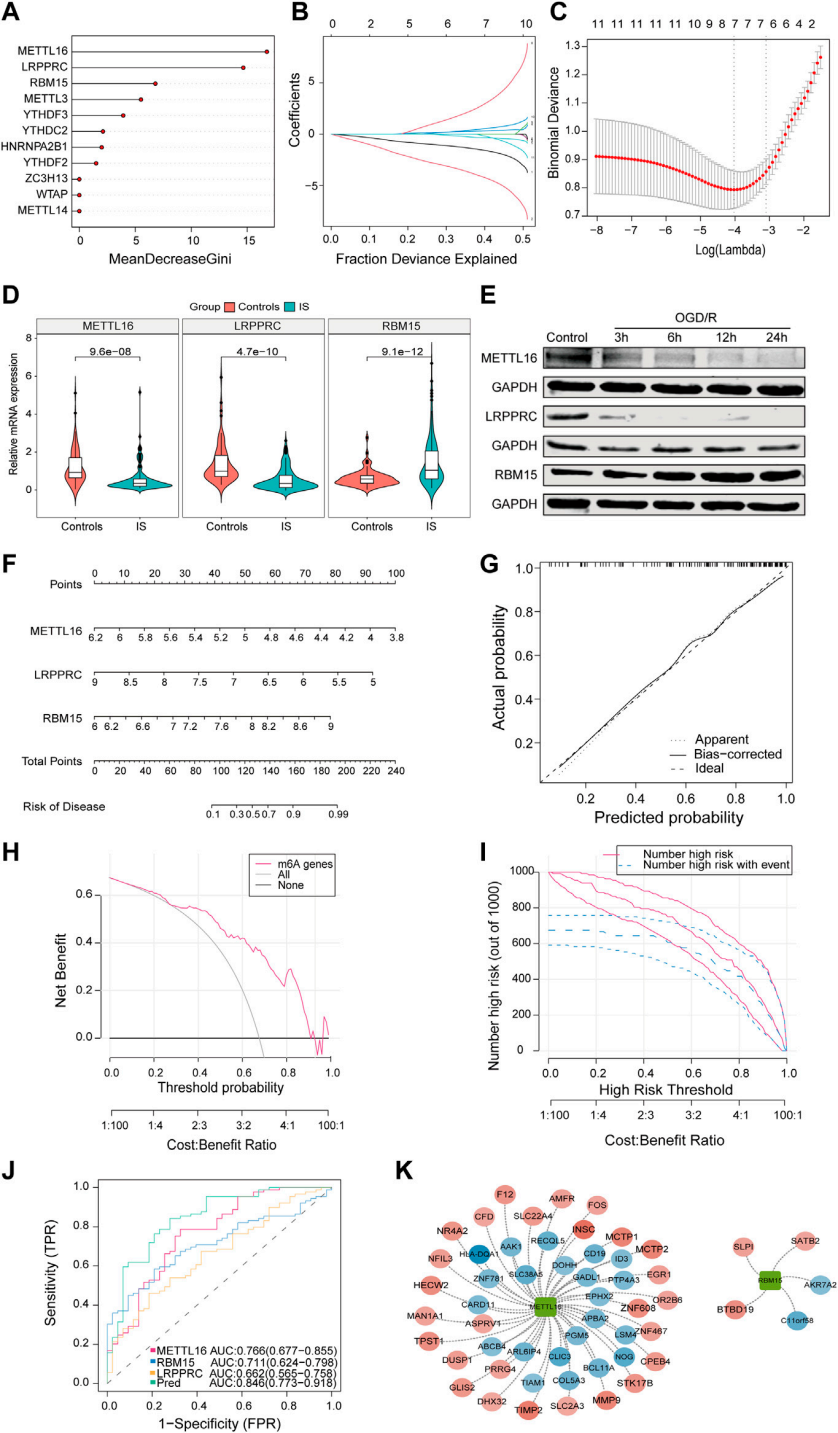
The characteristics of METTL16, LRPPRC, and RBM15 produced an ideal model with high accuracy. **(A)** The importance of the m6A regulators in the RF model. **(B)** LASSO coefficient profiles of m6A regulators. **(C)** LASSO regression tuning parameter selection using 10-fold cross-validation. **(D)** Expression of METTL16 (*p* = 9.6*e^−8^), LRPPRC (*p* = 4.7*e^−10^) and RBM15 (*p* = 9.1*e^−12^) in clinical samples. **(E)** Expression of three key regulators in the OGD/R model. **(F)** The risk scores of 3 m6A regulators. **(G)** Predictive ability of the nomogram model as revealed by the calibration curve. **(H)** DCA curve indicating benefit with respect to IS samples. **(I)** Clinical impact of the nomogram model as assessed by the clinical impact curve. **(J)** The discrimination ability of the three crucial m6A regulators for controls and IS samples was analyzed by ROC curve analysis and evaluated by AUC values. **(K)** Validation of cross-talk of three crucial m6A regulators and targets by PPI network analysis. AUC: area under the curve.

Next, we analyzed 11 differentially expressed m6A regulators. RF was used to rank genes according to their importance for visualization and six genes were identified (METTL16, LRPPRC, RBM15, METTL3, YTHDF3, and YTHDC2) (Figure 2A). Using the LASSO algorithm for feature selection and dimensionality reduction, seven genes (METTL16, HNRNPA2B1, RBM15, YTHDF2, WTAP, and METTL14) were determined to be crucial for IS (Figures 2B, C). After intersecting the results of the two algorithms, three key genes (METTL16, RBM15, and LRPPRC) were obtained (Supplementary Figure S2A). Additionally, by collecting clinical specimens and applying qRT-PCR, we identified that METTL16 and LRPPRC were downregulated and RBM15 was upregulated in IS samples (Figure 2D). Subsequently, the OGD/R model was constructed using HMC3 cells, and the same results were obtained at the protein level by immunoblotting (Figure 2E). Next, the nomogram, calibration curve, DCA curves, and area under the curve (AUC) all further confirmed the importance of these three key m6A regulators in IS (Figures 2F–J). The results showed that the characteristics of METTL16, LRPPRC, and RBM15 produced an ideal model with high accuracy, and the combined analysis showed that the AUC of the predicted IS incidence was >0.8. Furthermore, using the external dataset GSE198710 resulted in the same outcome (Supplementary Figure S2B). The m6A2Target database was used to identify the differential targets of the three m6A regulators and to construct a network diagram (Figure 2K).

### 3.3 m6A regulators are associated with immune characteristics of IS

**FIGURE 3:**
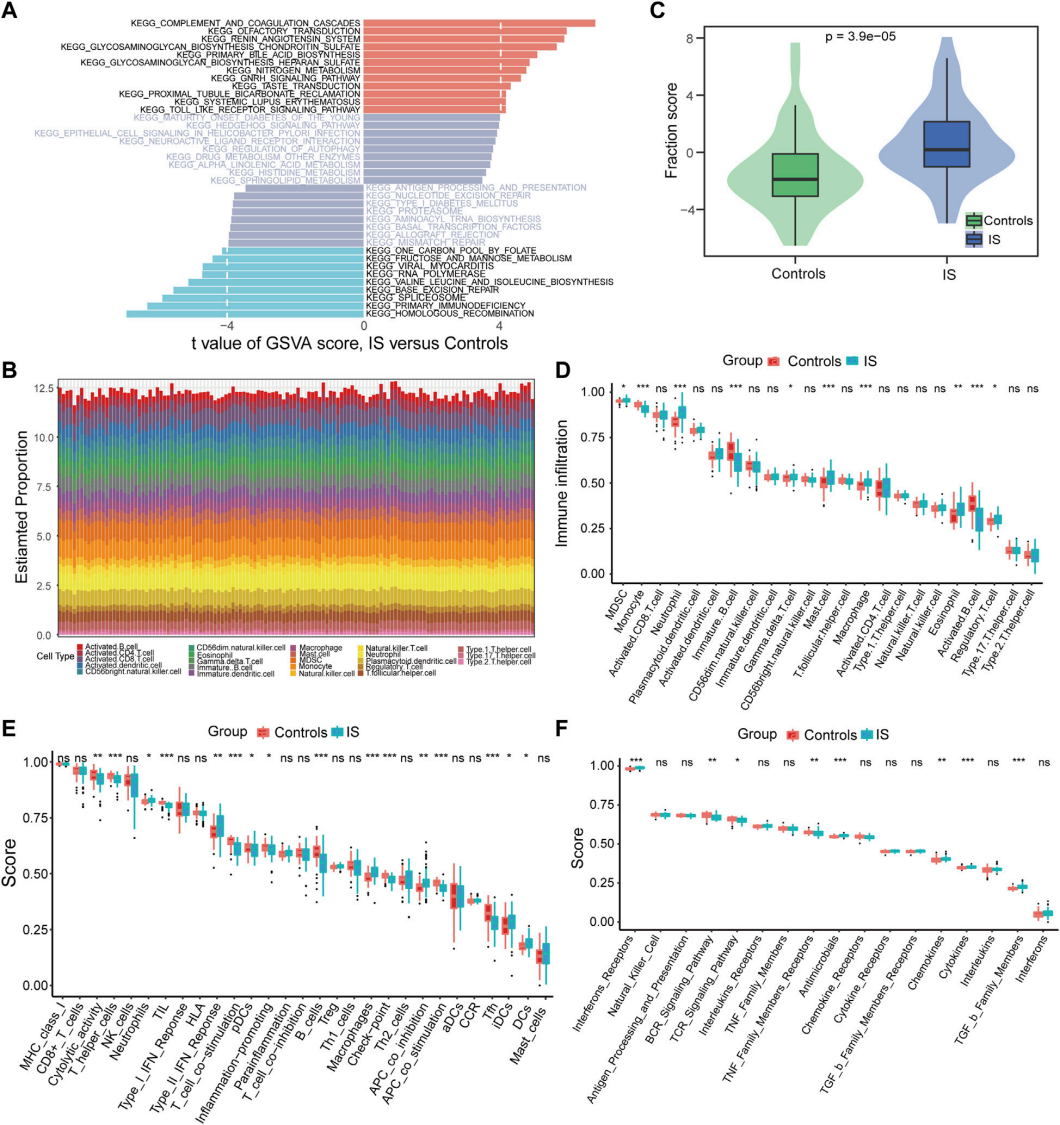
Differences in the immune microenvironment were identified between controls and IS samples. **(A)** Differences of KEGG pathway enrichment score. **(B)** Relative proportions of immune cell infiltration for each sample. **(C)** Differences of immune cell infiltration scores (*p* = 3.9*e^−5^). **(D–F)** Box plot of the abundance differences of immune microenvironment characteristics including infiltrating immune cells, immune functions, and immune responses, respectively. *p < 0.05, **p < 0.01, and ***p < 0.001, “ns” indicates no significance.

**FIGURE 4:**
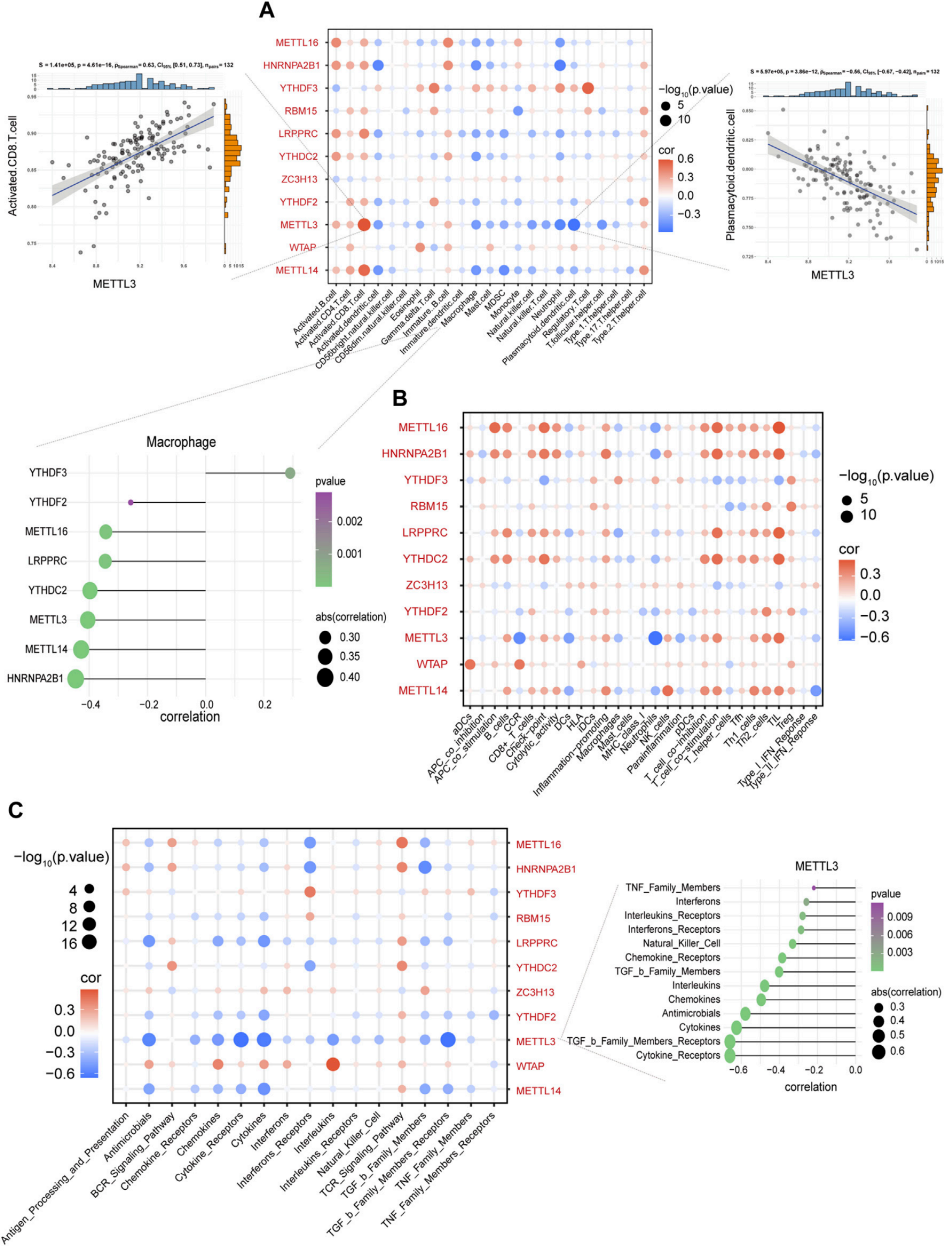
Macrophages were negatively correlated with most m6A regulators. **(A)** The correlation between immune cells and m6A regulators. METTL3 and activated CD8 T cells with the most negative correlation (left side scatter plot), and plasmacytoid dendritic cells with the most negative correlation (right side scatter plot). The lollipop plot presents macrophages and most m6A regulators with negative correlation. **(B)** The correlation between immune functions and m6A regulators. **(C)** The correlation between immune responses and m6A regulators. The lollipop plot presents METTL3 and most immune responses with negative correlation.

Using GSVA to compare the KEGG pathways between controls and IS samples, we found that the IS samples exhibited the highest enrichment of complement and coagulation cascades (Figure 3A). Based on the ssGSEA algorithm, we examined the expression of 23 immune cells (Figure 3B), 29 immune functions, and 17 immune responses in control and IS samples. The immune cell infiltration score was higher in IS samples than in control samples (Figure 3C). Among them, neutrophils, mast cells, macrophages, and eosinophils were significantly upregulated, whereas monocytes and B cells were significantly downregulated (Figure 3D). The correlation analysis revealed that macrophages were negatively correlated with most m6A regulators. Additionally, METTL3 was positively correlated with activated CD8 T cells and negatively correlated with plasmacytoid dendritic cells (Figure 4A). Regarding immune function, in the IS samples, except for macrophages, type II IFN response and antigen-presenting cell (APC) co-inhibition significantly increased, whereas T helper cells, TIL, T cell co-stimulation, B cells, checkpoints, and Tfh were significantly decreased (Figure 3E). Specifically, METTL16 had the highest positive correlation with TIL, and METTL3 had the highest negative correlation with neutrophils (Figure 4B). Regarding immune responses, IFN receptors, antimicrobials, cytokines, chemokines, and members of the TGF-β family were significantly higher in IS samples (Figure 3F). However, METTL3 was negatively correlated with most immune responses (Figure 4C). Furthermore, the correlation analysis showed that METTL16, HNRNPA2B1, LRPPRC, METTL3, and METTL14 were significantly correlated with the abovementioned differential immune infiltration phenotypes. Taken together, these findings suggest that IS significantly activates the immune response and is regulated by m6A regulators.

### 3.4 Consensus clustering of m6A modification patterns

**FIGURE 5:**
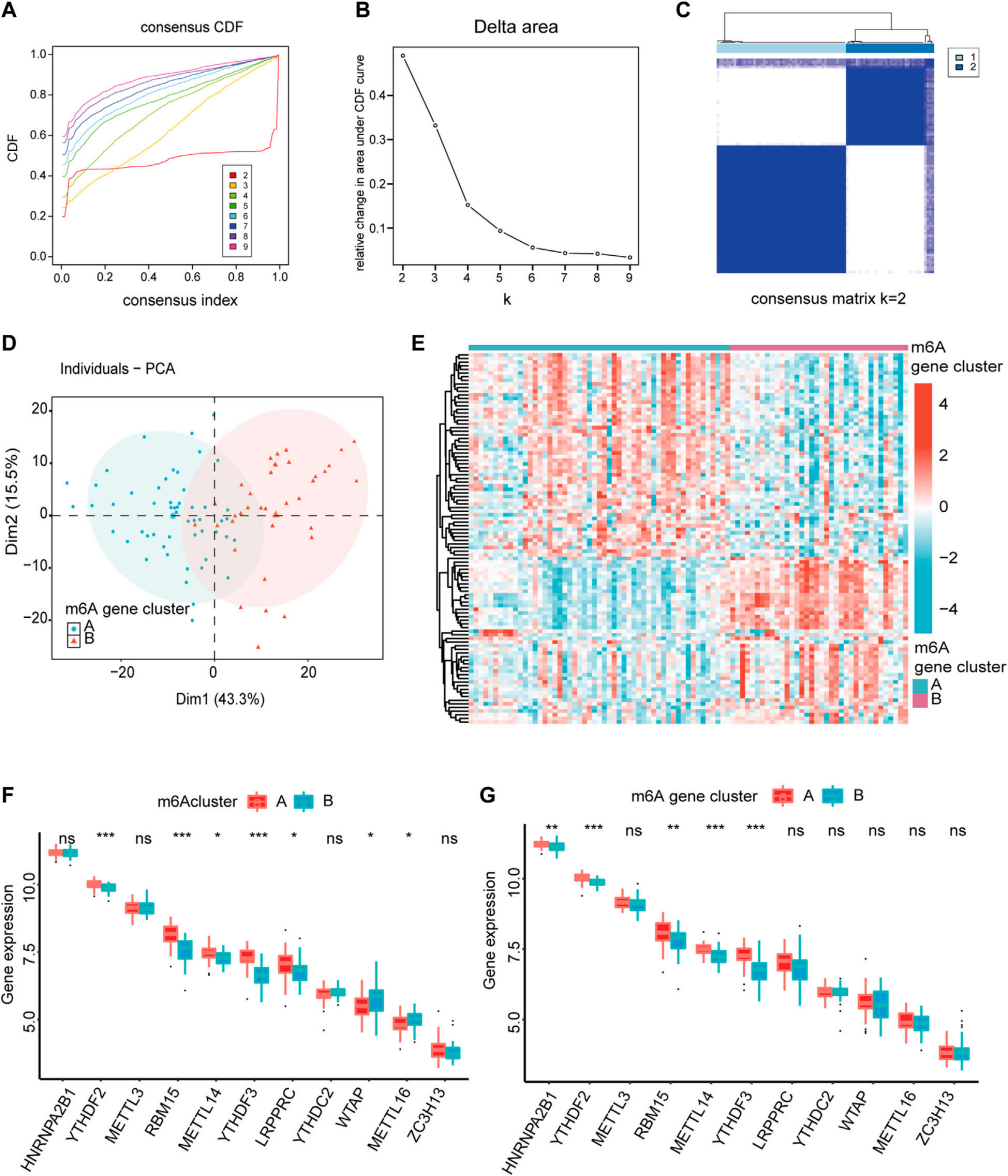
The heterogeneity of m6A regulators could be found in IS. **(A)** Consensus clustering CDF for k = 2–9. **(B)** Relative change in area under CDF curve fork = 2–9. **(C)** IS samples were divided into two m6A gene clusters when k = 2. **(D)** The PCA analysis for the expression profiles of two m6A gene clusters has few overlaps. **(E)** Expression heatmap of the 436 m6A-related DEGs in two m6A gene clusters. (red: high expression; blue: low expression). **(F,G)** The different expression status of m6A regulators between the two m6Aclusters and two m6A gene clusters. The lower and upper ends of the boxes represent the interquartile range of values. Outliers are represented by black dots in the boxes, and medians by lines. *p < 0.05, **p < 0.01, and ***p < 0.001, “ns” indicates no significance.

To analyze the heterogeneity of m6A regulators in IS, IS samples were classified into two m6A modification patterns by different m6A regulators, namely, m6A cluster A and B. In total, 102 m6A-related differentially expressed genes (DEGs) were identified and visualized using volcano plots based on the different m6A modification patterns (Supplementary Figure S3). Based on the obtained m6A-related DEGs, we divided the IS samples into different m6A gene modification patterns which were named as m6A gene cluster A and B (Figures 5A–C). PCA indicated significant differences in m6A expression profiles between the two distinct modification patterns (Figure 5D, Supplementary Figure S3D). This confirmed that there were two distinct methylation modification patterns in the IS. Figure 5E depicts the expression levels of the 436 m6A-related DEGs in m6A gene cluster A and B. The Wilcoxon test confirmed that the expression levels of m6A regulators differed between the two patterns (Figures 5F, G). Writers and readers showed significant differences between both m6A modification patterns, particularly YTHDF2 and YTHDF3, and their expression was decreased in m6A gene cluster B. In conclusion, m6A regulators form different m6A modifications in IS, which may be associated with the pathogenesis of IS.

### 3.5 Characteristics of the immune microenvironment under m6A modification patterns

**FIGURE 6:**
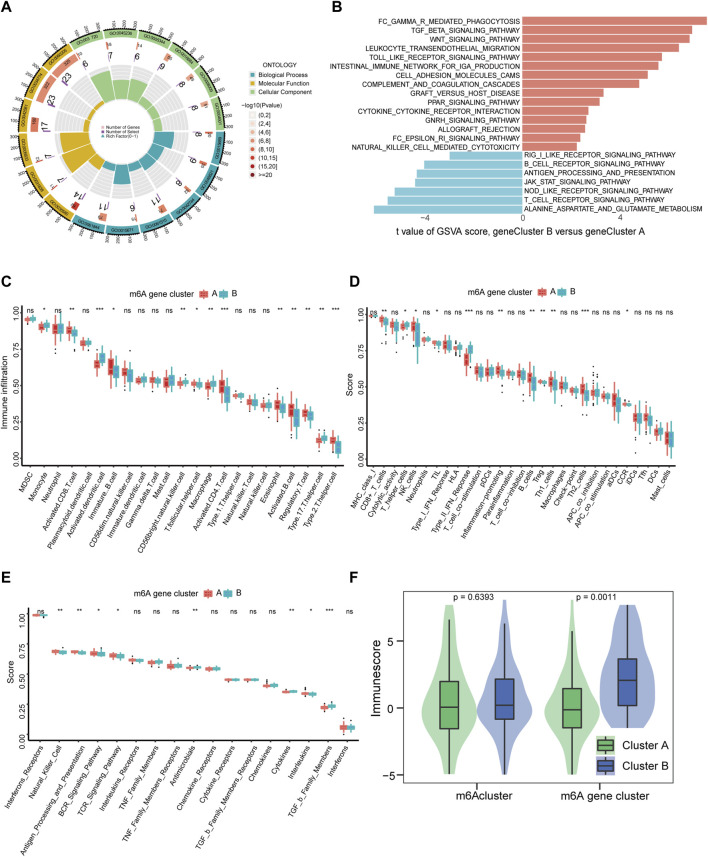
Immune signatures of m6A gene modification patterns. **(A)** GO functional analysis to explore the potential biological processes of 436 m6A-related DEGs for IS with enrichment circle plot. **(B)** Differences in KEGG pathway enrichment score. Upregulated pathways are shown in red and downregulated pathways are shown in blue. **(C–E)** Box plots of the differences in the abundance of immune cells, immune functions, and immune responses in each immune microenvironment, respectively. **(F)** Differences in immune cell infiltration scores between the two m6Aclusters (*p* = 0.6393) and two m6A gene clusters (*p* = 0.0011). *p < 0.05, **p < 0.01, and ***p < 0.001, “ns” indicates no significance.

Furthermore, we used GO functional analysis to illustrate the differences in biological behavior between m6A clusters and found that these m6A-related DEGs were mainly distinct in GO:0031720 and GO:0005833 for m6A gene clusters (Figure 6A, Supplementary Figure S4A). Interestingly, KEGG pathway enrichment analysis using the GSVA package revealed that m6A gene cluster A had more adaptive immune pathways, while m6A gene cluster B comprised more innate immune pathways, including Fcγ R-mediated phagocytosis, TGF-β signaling pathway, intestinal immune network promoting IgA production, transendothelial migration of leukocytes, and natural killer cell mediated cytotoxicity (Figure 6B). These results further support the hypothesis that m6A gene clusters are closely related to the immune response. Among the different m6A modifications, m6A cluster A was more enriched in immune and metabolic processes, while m6A cluster B was mainly enriched in the repair of DNA damage and base excision (Supplementary Figure S4B).

Increasing evidence indicates that m6A modifications contribute to tumor immunity (Song et al., 2021). Subsequently, to further elucidate the association between m6A modifications and the immune microenvironment in IS, we analyzed each m6A cluster using the ssGSEA algorithm. This outcome was consistent with GSVA data. In m6A cluster B, activated dendritic cells, macrophages, Th 17 cells, Th cells, type II IFN response, antimicrobials, cytokines, and receptors of TGF-β family members were significantly upregulated, and METTL3 and METTL14 were significantly correlated with them. Contrastingly, activated CD8 T cells, activated CD4 T cells, activated B cells, Th2 cells, Th1 cells, and Tregs were significantly downregulated, while METTL16, HNRNPA2B1, LRPPRC, and RBM15 were significantly positively correlated (Figures 6C–E, Supplementary Figure S4C–E). Additionally, m6A gene cluster B had a much higher immune cell infiltration score than m6A gene cluster A (Figure 6F). Further, compared with m6A cluster A, m6Acluster B was upregulated in monocytes and INF, and significantly downregulated in Th2 cells, CD8 T cells, Tregs, and chemokines (Supplementary Figure S4F–H). The abovementioned results demonstrate that the immune characteristics differed in different gene clusters and were closely correlated with m6A regulators.

### 3.6 m6A score construction

**FIGURE 7:**
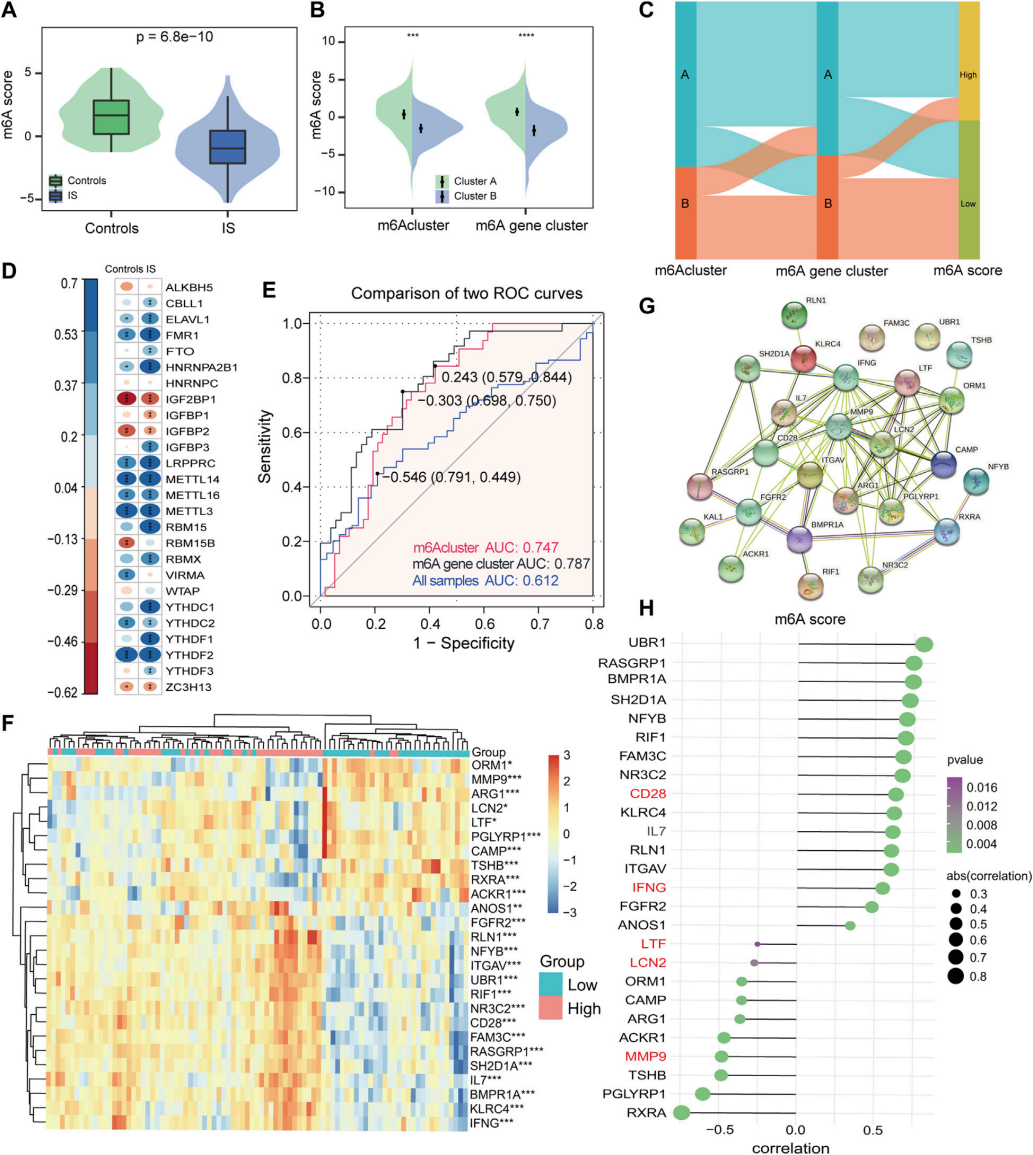
Construction of the m6A score signature. **(A)** Differences in m6A score between controls and IS samples (*p* = 6.8*e^−10^). **(B)** Differences in m6A score between two m6Aclusters and two m6A gene clusters. Wilcoxon test was applied to compare the statistical variations. **(C)** Sankey diagram showing the relationship among m6Aclusters, m6A gene clusters, and m6A score. **(D)** Correlations between m6A score and m6A regulators among controls and IS samples. **(E)** Discrimination ability of m6A score for both m6Aclusters and m6A gene clusters was assessed by AUC values. **(F)** Heatmap of IRs between high and low m6A score groups. **(G)** PPI network of IRs. **(H)** Correlation of IRs with m6A score. *p < 0.05, **p < 0.01, and ***p < 0.001, “ns” indicates no significance.

Consequently, considering the complexity and heterogeneity of m6A modifications, the PCA algorithm was utilized to calculate the m6A score for each sample. It was found that controls had a significantly higher m6A score compared with IS samples (Figure 7A), and m6A cluster A had a much higher m6A score than m6A cluster B in different subtypes of m6A modifications (Figure 7B). Furthermore, with 0 as the cut-off value, the m6A score was divided into high and low groups to determine the relationship among m6A clusters, m6A gene clusters, and m6A scores, visualized in a Sankey diagram (Figure 7C). To better characterize the m6A score, we evaluated the relationship between 26 m6A regulators and m6A scores, and found that the m6A score was significantly negatively correlated with RBM15 in IS, but not with RBM15 in controls (Figure 7D), once again demonstrating that RBM15 may play a significant role in IS. Figure 7E shows that the m6A score has good diagnostic potential for different subtypes of m6A modifications. Figure 7F displays a total of 26 IRs identified by comparing the results of the two groups (high vs. low m6A score) in the IS. From the string database, the IRs with the highest interaction relationship scores were CD28, IFNG, LTF, LCN2, and MMP9 (Figure 7G). Finally, the correlation analysis also revealed that the m6A score was significantly correlated with IRs (Figure 7H); this further confirms that m6A plays a non-negligible role in immune regulatory processes.

## 4 Discussion

Mounting evidence suggests that the immune system plays a dual role in IS pathophysiology (Iadecola et al., 2020). Several studies have found that m6A is an abundant RNA modification in the brain (Yen and Chen, 2021), and that its dysregulation can cause immune dysregulation, developmental defects, and tumor progression (Wang et al., 2020; Xu et al., 2020; Song et al., 2021). Thus, it is crucial to clarify how m6A modification affects immune regulation with respect to IS.

First, we compared the differences in expression profiles between the control and IS samples. Among the 26 m6A regulators, we found that five readers and six writers, especially YTHDF3 and METTL16, showed statistically significant differences, suggesting that they may play a role in IS pathophysiology. However, the two erasers (FTO and ALKBH5) identified in the current study were not found to be statistically significant. In Similar to the findings of AK et al., 24-h reperfusion after transient MCAO induced YTHDF3 mRNA expression compared to the sham (Mehta et al., 2019). Nevertheless, Xu et al. studied neurons treated with OGD/R and rat MCAO, and found that FTO levels were significantly decreased. Overexpression of FTO attenuates ischemia-reperfusion-induced neuronal damage, whereas knockdown of ALKBH5 aggravates it, suggesting that erasers may also be associated with IS (XK Mo Y et al., 2020). However, it should be noted that there may be differences between rodents and humans in this regard, necessitating further exploration. Further, we found not only that m6A regulators showed protein interactions or expression correlations, but also positive or negative correlations between m6A regulators, which demonstrates a dynamic m6A modification balance in patients with IS. Particularly, ELAVL1 was strongly correlated with ALKBH5 in controls, whereas ELAVL1 was more strongly correlated with FTO in patients with IS, indicating that m6A regulators influence the occurrence and development of IS, and that the interactions between m6A regulators in different samples were concentrated.

Second, machine learning methods are efficient ways to identify important features or variables associated with the outcomes of interest. Using the LASSO and RF models, we identified three important IS-related m6A regulators, namely, METTL16, RBM15, and LRPPRC. KE et al. found that the METTL16 protein controls cellular SAM levels and installs m6A on U6 small nucleic acids, MALAT1, XIST, and MAT2A (Pendleton et al., 2017). Nuclear METTL16 protein and cytoplasmic METTL16 protein play different roles in cellular transcription and translation (Su et al., 2022). Despite this, there has been no basic research on the role of METTL16 in immune regulation. Notably, RBM15 involvement in the immune regulation of M1 macrophages has been shown (Li et al., 2022b). Higher levels of RBM15 stimulate antigen-presenting cells, enhance inflammation, and upregulate major histocompatibility complex class I, Th2s, and Tregs (Tang et al., 2020). LRPPRC is generally considered an autophagy gene (Ruzzenente et al., 2011; Li et al., 2020). Most studies have found that LRPPRC is closely associated with neurodegenerative diseases, neurofibromatosis, venous thromboembolism, non-alcoholic fatty liver, and viral infections (Hosp et al., 2015; Cui et al., 2019) and that its dysregulation may initiate carcinogenesis and inhibit cancer cell apoptosis (Gu et al., 2021a). However, no research has been conducted on the effect of LRPPRC on immunity. Although how these three key genes regulate the pathogenesis of IS has not been reported, our findings, taken together, can be utilized to distinguish patients with IS from controls, which may prove useful for diagnosis in the future.

Third, the CNS immunity involves immune cell infiltration, migration, and activation (Zhang et al., 2019). When IS occurs, C1q deposition leads to the release of C3a and C5a, and the deposition of membrane attack complexes, resulting in persistent inflammation and brain damage (Mocco et al., 2006). Our findings were consistent with the fact that the IS group was most enriched in complement and coagulation cascades and had more active immune levels compared to controls (e.g., neutrophil activation and macrophage-mediated immunity involved in the immune response). Notably, our study found similar results. Macrophages are closely associated with m6A regulators, and METTL3 is crucial to the immune phenotype. When stimulated by lipopolysaccharides, YTHDF2-deficient macrophages can activate the MAPK and NF-κB signaling pathways and enhance the expression levels of signaling molecules, particularly TNF-α, IL-1β, IL-6, and IL-12 (Yu et al., 2019). METTL3-deficient macrophages suppress oxLDL-induced m6A levels and inflammatory responses (Quiles-Jiménez et al., 2022). m6A regulators also play a critical role in macrophage polarization. FTO was discovered to contribute in the same manner to M1 and M2 macrophage activation (Gu et al., 2020). However, previous studies have found that METTL3, METTL14, HNRNPA2B1, and FMR1 promote M1 macrophage polarization, and that METTL3 and IGF2BP3 inhibits M2 macrophage polarization. Contrastingly, WTAP and IGF2BP2 promoted M1 polarization in addition to inhibited M2 polarization (Liu et al., 2019; Liu et al., 2022). Furthermore, most studies have demonstrated that METTL3 overexpression attenuates lipopolysaccharide-induced inflammatory responses in NF-κB-dependent macrophages. Conversely, METTL3 overexpression accelerates the release of pro-inflammatory cytokines and inflammatory proteins (Gu et al., 2021b).

To further demonstrate the potential pathogenesis of IS, unsupervised clustering of IS samples was performed using m6A regulatory expression profiles. Therefore, two modification patterns were identified, including m6Aclusters and m6A gene clusters, which highlighted the role of m6A regulators in IS. There was greater immune infiltration in cluster B than in cluster A, but no statistical difference was observed between the m6Acluster subtypes. GSVA analysis was consistent with the ssGSEA results, revealing that m6A gene cluster A was associated with adaptive immunity and m6A gene cluster B with innate immunity. Previous studies have suggested that in the initial stages of IS, receptors on innate immune cells are first activated, and danger signals then stimulate the inflammasome, resulting in systemic immune activation. Subsequently, patients can develop severe immunosuppression. In the chronic stage, antigen presentation triggers adaptive immunity in the brain (Iadecola et al., 2020). Unfortunately, no immunotherapy against IS has yet been found. Thus, m6A gene modification patterns can help us understand the immune environment after IS occurrence and design novel anti-ischemic therapies. We also observed that METTL3, METTL14, METTL16, HNRNPA2B1, LRPPRC, and RBM15 are closely associated with the immune signature of IS. The introduction of METTL3, METTL16, LRPPRC, and RBM15 has been detailed before; therefore, they will not be repeated here. Although METTL14 lacks a SAM binding site and catalytic activity, METTL3 activity is highly reliant on METTL14, which is essential for complex integrity maintenance and the recognition of certain RNA substrates (Zhou et al., 2021). In a mouse model of colitis, Lu et al. reported that METTL14 deficiency enhanced cytokine production by Th1 and Th17 cells and inhibited Treg differentiation (Lu et al., 2020). Furthermore, METTL14 had a significant effect on cell development. Inactivating METTL14 not only reduces oligodendrocyte numbers and CNS myelination (Xu et al., 2020) but also severely impairs B cell development and affects embryonic stem cell self-assembly (Zheng et al., 2020). During viral infection, HNRNPA2B1 functions as a reader protein that detects viral DNA and induces m6A modification to initiate an innate immune response (Wang et al., 2019b). As early m6A-related studies on IS were limited and focused on single cell types, a comprehensive analysis of m6A regulators enables an improved understanding of the molecular features and inherent immune status of IS with high heterogeneity. The two m6A modification patterns of IS can be considered as a molecular-level classification, which helps understand its pathogenesis from the perspective of m6A modification as well as the underlying immunomodulatory mechanisms.

Finally, we compared the m6A score with immune genes in the high and low groups. There was a positive correlation between the m6A score and CD28 and IFNG, as well as a negative correlation between the m6A score and LTF, LCN2, and MMP9. For instance, CD28 is essential for helper T cell type 2 development, as well as for AKT and TCR signaling in naive CD4^+^ T cells (Esensten et al., 2016). IFNG has antiviral and immunomodulatory properties and promotes Th1 cell differentiation by regulating the JAK-STAT pathway and upregulating the transcription factor T-bet (DA SK Hertzog et al., 2004). LTF is one of the most abundant proteins secreted by neutrophils with immune and neural stem cell functions (Wang et al., 2017). LCN2 stimulates the synthesis of chemokines in the CNS in response to neuroinflammatory, and is actively implicated in innate immune responses (Luo et al., 2022). The primary mechanism of action of MMP9 in brain disorders appears to be its involvement in host defense, as well as its contribution to blood-brain barrier disruption (Vafadari et al., 2016; Liu et al., 2020). Therefore, consistent with the previous description, the high m6A score group (m6A gene cluster A) was more enriched in the T cell immune response, while the low m6A score group (m6A gene cluster B) was enriched in innate immunity. The combination of m6A score can determine the methylation modification patterns of different subtypes and help clarify the immune process of IS.

There are limitations to this study. First, based on the bioinformatics analysis in this study, single-cell sequencing or even multi-omics is needed to validate the m6A methylation mechanism, despite our validation of differential expression of key molecules in clinical samples and cellular models. Second, peripheral blood samples were used, and no detailed clinical data on patients were obtained; therefore, it was difficult to reveal the role of m6A modification in immune regulation from multiple viewpoints. Hopefully, the first-hand analysis of tissue samples and follow-up patient information will provide more helpful information in the future. Immune cell analysis also uses the most prevalent analytical methods to quantify immune cell numbers; however, single-cell sequencing is still required to obtain the most precise results. Our findings confirm that m6A modification strongly influences immune properties, providing new insights into the pathogenesis, pathophysiology, and clinical phenotypes of IS. The following gaps remain in our research: retrospective studies exhibit statistical bias, lack patient survival information, and comprehensive exploration of individual heterogeneity. Therefore, further prospective studies are necessary to obtain a better fit. Non-etheless, we have developed a superior prediction model based on m6A modification with good applicability, which allows us to inexpensively quantify the immune profile of patients with IS.

## 5 Conclusion

In summary, this study identified three key m6A regulators and two different m6A epigenetic modification patterns. Based on transcriptional expression data, we integrated m6A-regulated signature genes and quantitative methods to assess immune cells, immune functions, and immune responses, and determined the relationship between m6A modifications and immune characteristics. The heterogeneity and complexity of patients with IS may be partly explained by the differences in m6A modification patterns. This is the first study to examine RNA methylation modifications and immune microenvironment characteristics in ISs using the m6A regulatory mechanism. The use of m6A modification opens a new chapter in understanding the immune-brain relationship and encourages future studies to actively investigate the anti-ischemic response to immunomodulatory therapy in clinical practice.

## Data Availability

The original contributions presented in the study are included in the article/Supplementary Material, further inquiries can be directed to the corresponding authors.
