# Supplementary material for: RNA methylation pattern and immune microenvironment characteristics mediated by m6A regulator in ischemic stroke
Source: Front Genet. 2023 Apr 17;14:1148510. doi: 10.3389/fgene.2023.1148510 (PMC10150022; doi:10.3389/fgene.2023.1148510)
Supplement: Supplementary file 1 [file DataSheet1.docx]

**RNA methylation pattern and immune microenvironment characteristics mediated by M6A regulator in ischemic stroke**

**Kejuan Jia^1,2^, Wenbo Xia^1,2^, Qian Su^1,2^, Shiqi Yang^1^, Yanli Zhang^1^, Xunran Ni^3^, Zhiqiang Su^1^, and Delong Meng^1*^**

# Supplementary Material


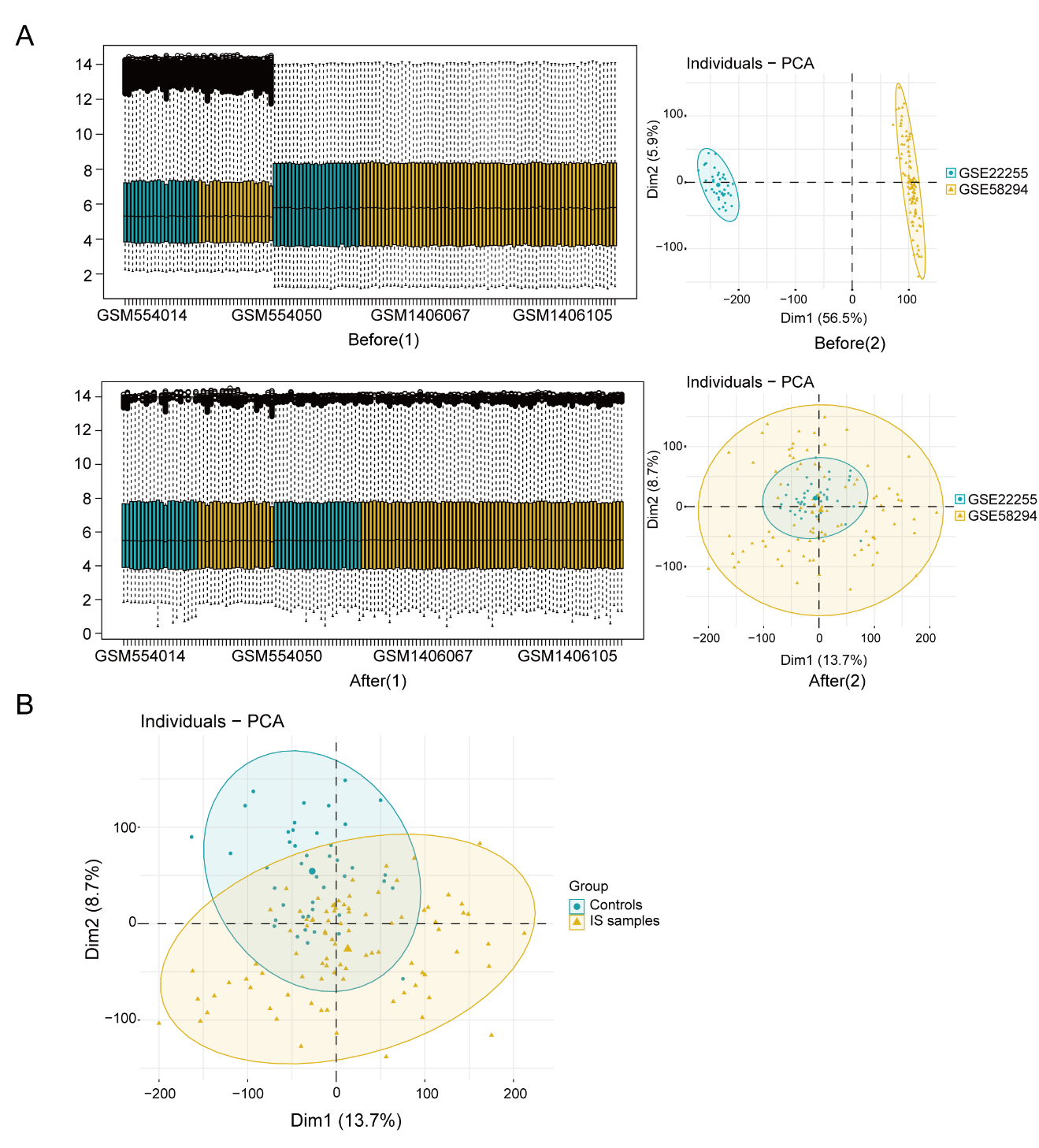


**Figure S1** (A) The box plot and the PCA plot of GSE22255 and GSE58294 datasets before and after the batch effect removal. (B) The PCA plot of the GSE22255 and GSE58294 datasets after sample correction.


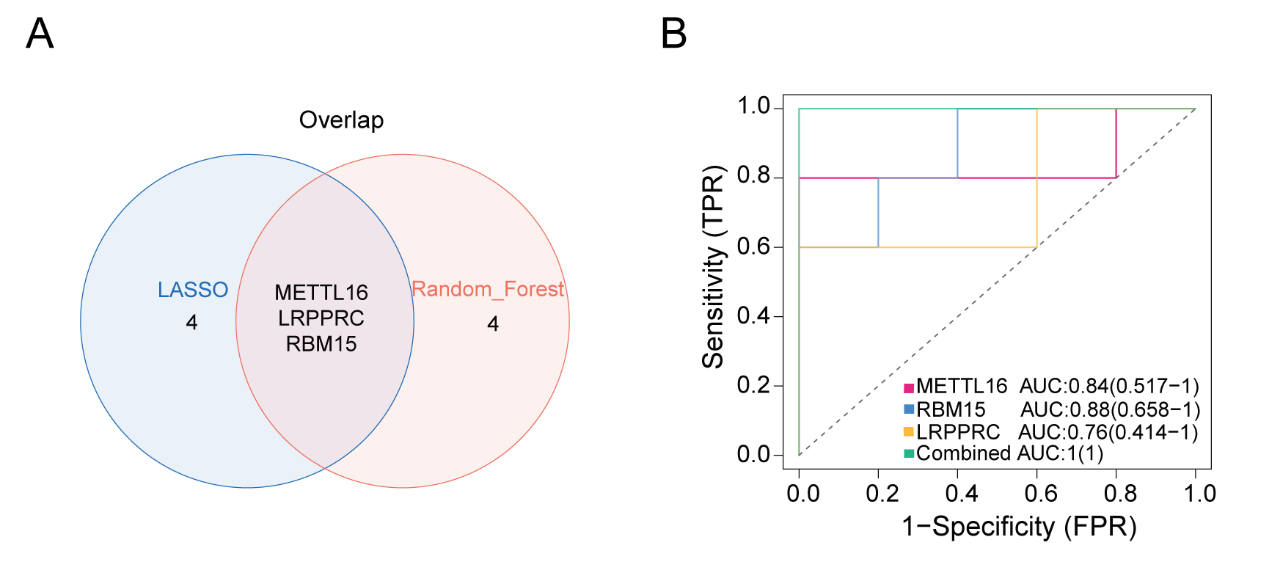


**Figure S2** (A) Venn diagram demonstrating three key m6A regulators shared by the LASSO and RF algorithms. (B) The discrimination ability of the three key m6A regulators was evaluated by ROC curve and AUC value based on the validation set GSE198710.


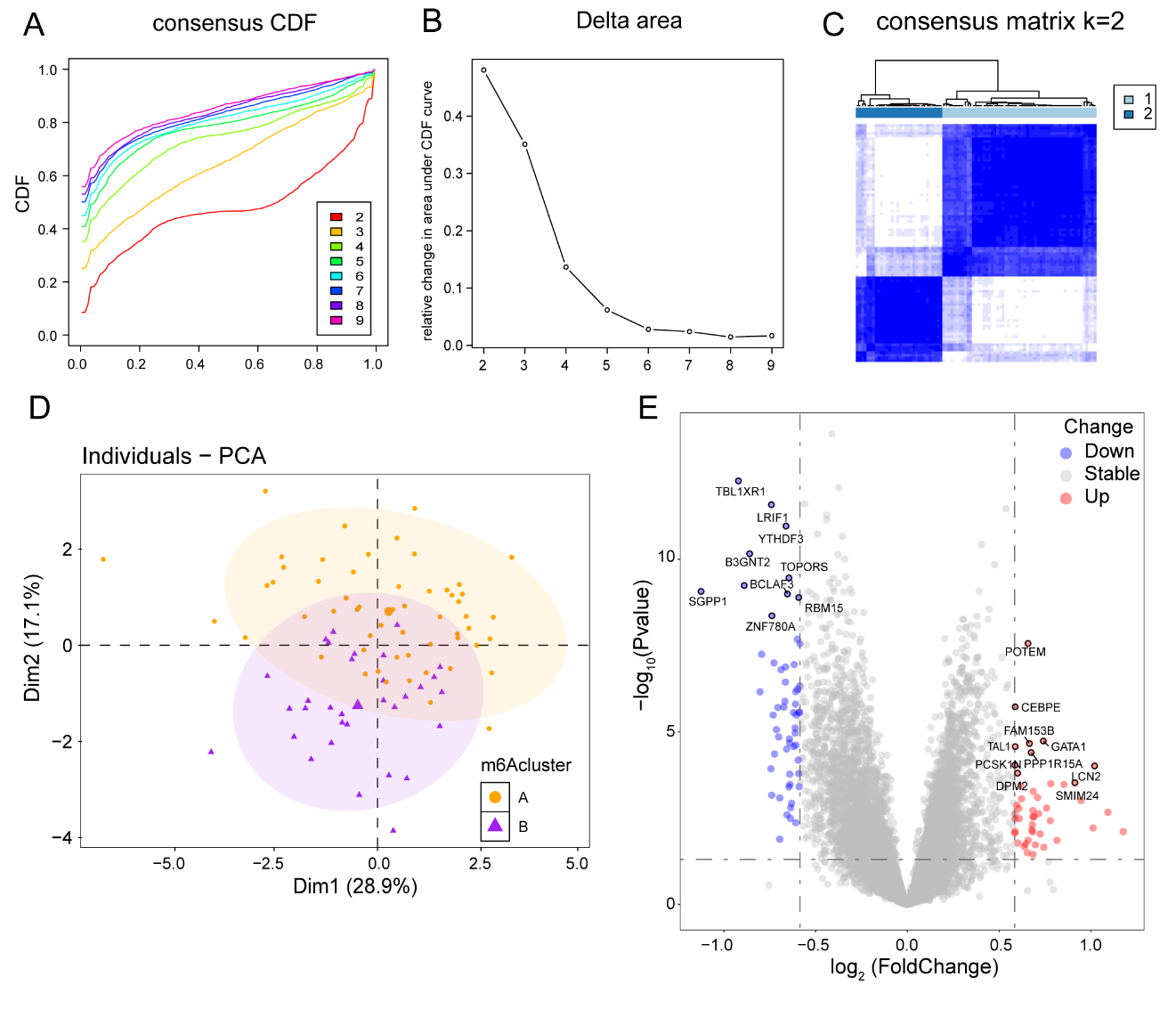


**Figure S3** Consensus clustering of m6A modification patterns. (A) Consensus clustering CDF for k = 2–9. (B) Relative change in area under CDF curve fork = 2–9. (C) IS samples were divided into two m6Aclusters when k = 2. (D) The PCA analysis for the expression profiles of two m6Aclusters has few overlaps. (E) Volcano plot of m6A-related DEGs between two m6Aclusters.


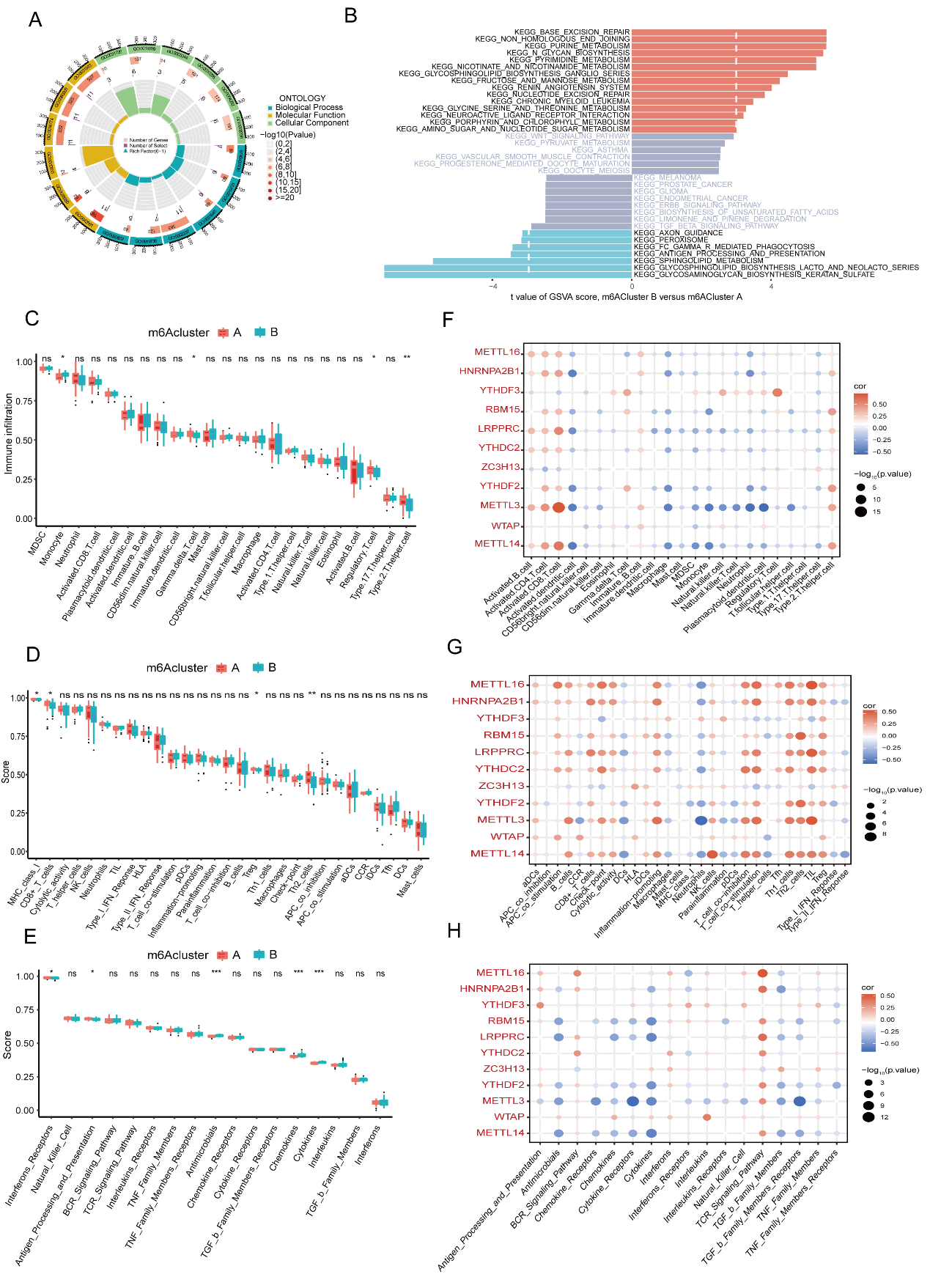


**Figure S4** Immune signatures of m6A modification patterns and correlation between immune infiltration microenvironment and m6A regulators. (A) GO functional analysis to explore different m6Aclusters with enrichment circle plot. (B) Differences of KEGG pathway enrichment score. (C, D, and E). Correlation of each immune microenvironment with m6A regulators, including immune cells, immune functions, and immune responses. (F, G, and H) Box plots of the differences in the abundance of immune cells, immune functions, and immune responses in each immune microenvironment, respectively. * p <0.05, ** p <0.01, and *** p <0.001, “ns” indicates no significance.
